# Evaluating the safety of glucagon-like peptide-1 agonist use in non-diabetic overweight patients undergoing shoulder arthroplasty

**DOI:** 10.1016/j.jsea.2026.100088

**Published:** 2026-09-09

**Authors:** Amelia Hummel, Alexander J. Acuña, Alexander Hornung, Ryan Lew, William Harkin, Grant E. Garrigues, Gregory Nicholson

**Affiliations:** Department of Orthopedic Surgery, Rush University Medical Center, Chicago, IL, USA

**Keywords:** Total shoulder arthroplasty (TSA), Reverse total shoulder arthroplasty (rTSA), Glucagon-like peptide 1 agonist (GLP-1 agonist), Obesity, Complications, Joint replacement, shoulder, semaglutide

## Abstract

**Background:**

Initially indicated for diabetes mellitus (DM), glucagon-like peptide 1 (GLP-1) agonists are now indicated for obesity management in isolation of DM. While prior studies have examined post-operative outcomes after total shoulder arthroplasty (TSA) in GLP-1 users with DM, there is a paucity of literature evaluating its use in non-diabetic patients for weight loss rather than glucose control. Therefore, our analysis compared 90-day and 2-year outcomes following TSA between non-diabetic obese GLP-1 users and a matched cohort of obese GLP-1 non-users.

**Methods:**

The PearlDiver Mariner database was queried to identify patients who underwent anatomic TSA and reverse TSA between 2010 and 2023. Patients with a history of DM or who underwent TSA for cancer, infection, or pathologic fractures were excluded. Patients with a body mass index ≥ 30 kg/m^2^ within 6 months of surgery were included. GLP-1 users (within 3 months of surgery) were matched in a 1:10 ratio to GLP-1 non-users by age, sex, Elixhauser Comorbidity Index, smoking status, and various comorbidities. Multivariate logistic regression models were utilized to compare outcomes at 90 days and 2 years.

**Results:**

After matching, 462 GLP-1 users and 4,377 GLP-1 non-users were included in our analysis. GLP-1 users had higher readmission rates (odds ratio: 2.034; 95% confidence interval: 1.594-2.578; *P* < .001) and subsequently overall complication rates (odds ratio: 1.474; 95% confidence interval: 1.295-1.799; *P* < .001) compared to GLP-1 non-users at 90 days. However, no differences were seen between cohorts for the remaining 90-day outcomes nor for all evaluated 2-year outcomes including prosthetic joint infection, fracture, and all-cause revision.

**Discussion:**

Our analysis found a higher incidence of overall complications at 90 days among obese, non-diabetic GLP-1 users, driven largely by an increased incidence of readmissions. However, there were largely comparable outcomes at 2 years. These findings suggest that while GLP-1 agonists may be reasonable to use in the perioperative period, interventions to reduce readmissions among these patients may be warranted.

As the obesity epidemic within the United States continues, there has been a growing incidence of end-stage shoulder osteoarthritis requiring surgical intervention.^6^^,^^13^ An abundance of data has shown that pre-operative obesity has been associated with worse outcomes after total shoulder arthroplasty (TSA).^12^ Specifically, obese patients tend to have higher rates of infection, medical complications, wound healing issues, and revision relative to normal weight controls.^2^^,^^5^^,^^9^ Subsequently, shoulder surgeons have continued to evaluate safe and effective methods of mitigating the effects of obesity on post-operative outcomes.

Initially indicated for diabetes mellitus (DM), glucagon-like peptide-1 agonists (GLP-1 agonists) are now indicated for obesity management in isolation of DM. Prior studies have examined post-operative outcomes after shoulder arthroplasty in diabetic GLP-1 users with mixed results.^3^^,^^7^^,^^11^ Although Seddio et al^11^ found a lower incidence of various adverse events including surgical site infection (SSI), venous thromboembolism, and emergency department visits among diabetic semaglutide users, Megerian et al^7^ reported comparable rates of both 90-day and 2-year complications between GLP-1 users and matched controls. In addition to the variable results reported in the literature, no prior study has evaluated the impact of GLP-1 agonists for weight loss rather than for glycemic control prior to TSA. As the use of GLP-1 agonists prior to TSA continues to rise, further evaluation of its impact on perioperative outcomes among non-diabetic patients with shoulder osteoarthritis remains essential.

In light of the ongoing obesity epidemic, medication-assisted weight loss has been increasingly utilized. There is a paucity of data evaluating the use of GLP-1 agonists for weight loss rather than for glycemic control prior to orthopedic procedures. Therefore, our analysis compared perioperative outcomes between non-diabetic obese patients using GLP-1s prior to TSA with a matched cohort of GLP-1 non-users. Specifically, we evaluated 90-day and 2-year outcomes following primary TSA.

## Methods

### Utilized database

The present analysis utilized the all-payer PearlDiver Mariner (PearlDiver Technologies, Fort Wayne, Indiana) administrative claims database.^1^ This claims database includes information on episodes of care for over 170 million patients across the United States. Information regarding surgical procedures, patient demographics, and perioperative complications is available through the use of Current Procedural Terminology, International Classification of Diseases (ICD), Ninth and ICD 10th Revision codes. Institutional review board approval was not required for the present study as all included patient records are de-identified.

### Patient selection

Patients undergoing elective anatomic TSA (aTSA) or reverse TSA) between 2010 and 2023 were identified by the Current Procedural Terminology, ICD Ninth, and ICD 10th codes outlined in Supplementary Appendix A. Patients with a body mass index ≥30 kg/m^2^ within 6 months of surgery and who received a prescription for a GLP-1 agonist within 3 months of surgery were included. GLP-1 agonist prescriptions were identified utilizing the Uniform System of Classification prescription codes 39251 and 18120. Patients were excluded if they underwent TSA for fracture, infection, or cancer. Similarly, patients with a history of diabetes as well as those without at least 2-year follow-up within the database were excluded.

GLP-1 users were matched in a 1:10 ratio to GLP-1 non-users based on age, Elixhauser Comorbidity Index, gender, smoking status, type of arthroplasty (aTSA and reverse TSA), and the following comorbidities: osteoporosis, chronic obstructive pulmonary dysfunction, congestive heart failure, smoking, chronic kidney disease, depression, hypothyroidism, coagulopathy, cancer, anemia, liver disease, and alcohol dependency. Balance between groups was confirmed using chi-squared and *t*-tests.

### Outcome measures

Primary outcomes evaluated included 90-day complications as well as 2-year outcomes data for the matched cohort. Ninety-day complications included surgical site infections (SSI), wound disruptions, prosthetic joint infections, deep vein thrombosis, pulmonary embolism, venous thromboembolism, hematoma, pneumonia, urinary tract infection, acute kidney injury, cardiac arrest, reoperation, death, readmissions, emergency department visit, and transfusions. Two-year outcomes included periprosthetic fractures, dislocation events, post-operative stiffness, loosening, osteolysis, and all-cause revisions.

### Statistical analysis

Chi-squared tests were implemented for comparisons of categorical variables, while Student’s *t*-tests were used for continuous variable comparisons. For each evaluated outcome, a multivariate logistic regression controlling for sex, Elixhauser Comorbidity Index, age, smoking status, osteoporosis, and arthroplasty type was performed. Odds ratios (ORs) with their respective 95% confidence intervals (95% CIs) were subsequently generated for each comparison. The statistical program R version 3.6.0 (R Foundation for Statistical Computing, Vienna, Austria) within the PearlDiver software was utilized for statistical analyses. A *P* value <.05 was considered significant.

## Results

### Patient demographics

In total, 589 (382 females, 207 males) patients taking GLP-1s and 49,222 (29,717 females, 19,505 males) patients who were not taking GLP-1s met the inclusion criteria. After matching, 462 GLP-1 users (321 females, 141 males) and 4,377 GLP-1 non-users (3,064 females and 1,313 males) were included in the final analysis (Table I). Cohorts were appropriately matched across all demographics in the matching algorithm (all *P* values >0.05).

**Table I:** Patient demographics

	GLP-1 users	GLP-1 non-users
Total (n)	462	4,377
Female	321	3,064
Male	141	1,313
Mean age (SD)	65.32 (6.57)	65.54 (6.38)
Mean ECI (SD)	6.36 (2.52)	6.30 (2.46)
Comorbidities		
Smokers	83	732
CKD	31	385
Depression	101	1,351
Hypothyroidism	99	1,092
COPD	55	622
CHF	22	247
Coagulopathy	12	195
Cancer	84	867
Anemia	68	771
Liver disease	25	303
EtOH	4	111

*COPD*, chronic obstructive pulmonary dysfunction; *CHF*, congestive heart failure; *CKD*, chronic kidney disease; *EtOH*, alcohol; *GLP-1*, glucagon-like peptide-1; *ECI*, Elixhauser Comorbidity Index; *SD*, standard deviation.

### Ninety-day outcomes

GLP-1 users had higher readmission rates (OR: 2.034; 95% CI: 1.594-2.578; *P* < .001) and subsequently overall complication rates (OR: 1.474; 95% CI: 1.295-1.799; *P* < .001) compared to GLP-1 non-users at 90 days (Table II). However, there were no differences in the remaining evaluated outcomes within 90 days of TSA.

**Table II:** Complications at 90 days and 2 years for GLP-1 users and GLP-1 non-users

Outcome	GLP-1 users	GLP-1 non-users	*P* value	OR (95% CI)	*P* value
90-d outcomes					
SSI	2 (0.43)	42 (0.96)	.381	0.425 (0.069-1.39)	.239
PJI	2 (0.43)	12 (0.27)	.882	1.469 (0.227-5.440)	.617
DVT	7 (1.52)	75 (1.71)	.901	0.859 (0.357-1.749)	.703
PE	4 (0.87)	63 (1.44)	.427	0.567 (0.170-1.383)	.272
Combined VTE	10 (2.16)	120 (2.74)	.563	0.755 (0.368-1.383)	.401
AKI	10 (2.16)	124 (2.83)	.494	0.709 (0.344-1.305)	.308
Cardiac arrest	0 (0)	9 (0.21)	.683	N/A	.991
Wound dehiscence	1 (0.22)	25 (0.57)	.511	0.370 (0.021-1.759)	.331
Hematoma	0 (0)	12 (0.27)	.525	N/A	.991
Nerve injury	1 (0.22)	3 (0.07)	.841	2.804 (0.137-22.461)	.377
Pneumonia	7 (1.52)	111 (2.54)	.232	0.569 (0.239-1.145)	.153
Transfusion	2 (0.43)	39 (0.89)	.450	0.459 (0.074-1.510)	.285
UTI	39 (8.44)	333 (7.61)	.300	1.102 (0.763-1.551)	.591
Death	0 (0)	1 (0.02)	1.000	N/A	.999
Reoperation	3 (0.65)	9 (0.21)	.183	3.005 (0.661-10.192)	.102
ED visit	93 (20.13)	827 (18.89)	.561	1.051 (0.819-1.337)	.692
Readmission	107 (23.16)	571 (13.05)	**<.001**	2.034 (1.594-2.578)	**<.001**
Any complication	203 (43.94)	1,514 (34.59)	**<.001**	1.474 (1.205-1.799)	**<.001**
2-y outcomes					
PJI	5 (1.08)	28 (0.64)	.423	1.573 (0.529-3.789)	.357
Fracture	17 (3.68)	134 (3.06)	.558	1.205 (0.694-1.964)	.479
Loosening	7 (1.52)	99 (2.26)	.381	0.644 (0.269-1.299)	.265
Dislocation	8 (1.73)	106 (2.42)	.442	0.703 (0.313-1.363)	.341
Osteolysis	1 (0.22)	13 (0.3)	1.000	0.698 (0.038-3.524)	.730
Revision	12 (2.6)	155 (3.54)	.356	0.710 (0.370-1.235)	.260

*SSI*, superficial surgical site infection; *DVT*, deep vein thrombosis; *PE*, pulmonary embolism; *VTE*, venous thromboembolism; *AKI*, acute kidney injury; *UTI*, urinary tract infection; *PJI*, periprosthetic joint infection; *GLP-1*, glucagon-like peptide-1; *ED*, emergency department.

### Two-year outcomes

There were also no significant differences between GLP-1 users and GLP-1 non-users for 2-year outcomes including prosthetic joint infections, fractures, loosening, dislocations, and osteolysis (Table II). Similarly, there was a comparable incidence of all-cause revision at 2 years (OR: 0.710; 95% CI: 0.370-1.235; *P* = .260).

## Discussion

As the use of GLP-1s among obese patients undergoing TSA continues to grow, further information is needed to assess the safety and potential benefits of these drugs during the perioperative period. Our analysis utilized a large, all-payer database to evaluate the impact of these medications on outcomes at both 90 days and 2 years among non-diabetic obese TSA patients relative to matched non-user controls. We found a higher incidence of both readmission and any complication among patients utilizing GLP-1s pre-operatively. However, no differences were seen for the remaining outcomes at 90 days or between cohorts at 2 years post-operatively.

Previous studies evaluating GLP-1 use among TSA patients have reported conflicting results. In their analysis of 518 GLP-1 agonist users undergoing TSA, Megerian et al^7^ reported a non-significant trend toward an increased incidence of acute kidney injury at 90 days and a decreased incidence of rotator cuff tear at 2 years following aTSA. The authors reported no significant differences at 90 days or 2 years following surgery. Conversely, Lawand et al^4^ found a higher incidence of multiple medical complications including myocardial infarction (OR: 2.84; 95% CI: 1.4-5.9; *P* = .003), pneumonia (OR: 2.225; 95% CI: 1.3-3.9; *P* = .003), and deep vein thrombosis (OR: 3.0; 95% CI: 1.5-5.9; *P* = .003) among GLP-1 receptor agonists relative to controls. However, Seddio et al^11^ found a lower incidence of these complications in addition to a lower incidence of urinary tract infection and SSI^77^. The conflicting findings among the current literature highlight the importance of further study into the impact of these medications on perioperative outcomes following TSA. Importantly, as all these studies primarily focused on diabetic patients utilizing this medication, our analysis adds to the literature by isolating those receiving GLP-1s for weight management.

Interestingly, the finding that GLP-1 agonists are associated with an increased incidence of readmission has previously been reported. In their analysis of the TriNetX database, Lawand et al^4^ reported a higher incidence of this complication (OR: 1.6; 95% CI: 1.2-2.2; *P* = .004) among GLP-1 users relative to propensity-score matched non-user controls undergoing TSA.^6^

While we were unable to elucidate the reasons behind the identified readmissions, it is possible that the gastrointestinal side effects of these medications may have contributed. Specifically, as recommendations related to perioperative discontinuation of GLP-1s to reduce anesthesia risks continue to change,^10^ associated readmission may be related to side effects seen at medication reinitiation.

A majority of previous studies have similarly found that GLP-1 agonist use does not impact the incidence of complications at 2 years post-operatively. In a recent systematic review of 6 studies and 43,415 patients undergoing shoulder surgery (including TSA), Moews et al^8^ found that GLP-1 agonist use was not associated with adverse events at 2 years. Similarly, Lawand et al^4^ and Megerian et al^7^ found comparable rates of revision at 2 years between either GLP-1 user or non-user cohorts. These findings in conjunction with the relatively comparable 90-day outcomes shown in our analysis suggest that GLP-1 use should not be a contraindication to TSA, and in the appropriately selected patient, may be safely utilized.

Our analysis has some limitations. Given the inherent limitations of the utilized database, we were unable to evaluate how factors such as surgeon experience, implant choice, and surgical setting may have impacted the evaluated outcomes. Similarly, as GLP-1 use for obesity in the absence of DM is a relatively new indication for its use, there were a limited number of patients available within the GLP-1 user cohort, which may limit our ability to detect differences in less common complications. Additionally, we were unable to evaluate possible other interventions being implemented to reduce the incidence of complications among included obese patients or to help these patients pre-operatively lose weight. Furthermore, although we found an increased incidence of readmission among this patient population utilizing GLP-1s, we were unable to identify the cause of each readmission and therefore are unable to determine whether it was directly associated with medication use or an additional confounding factor, which is another limitation of the available data.

## Conclusion

Our analysis found a higher incidence of overall complications at 90 days among obese non-diabetic GLP-1 users, driven largely by an increased incidence of readmissions. However, there were largely comparable outcomes at 2 years. These findings suggest that while GLP-1 agonists may be reasonable to use in the perioperative period, interventions to reduce readmissions among these patients are warranted. Future research is needed to better understand the factors contributing to the observed readmission rates among GLP-1 users, as well as the long-term effects of these medications.

## Disclaimers:

Funding: No funding was disclosed by the authors.

Conflicts of interest: Grant E. Garrigues has the following disclosures: Consultancy: Enovis, Tornier, and Mitek. Royalties: Enovis and Tornier. Stock: Aevumed, Patient IQ, and ROM 3; and is a designer of both an augmented anatomic glenoid and a reverse total shoulder.

Gregory Nicholson has the following disclosures: Royalties: Wright Medical Technology, Inc., Innomed, and Arthrosurface. Consultancy: Wright Medical Technology, Inc. Research support: Arthrex, Inc. and Wright Medical Inc. Fellowship education support: Wright Medical Inc. and Smith & Nephew. Paid Presenter: Arthrosurface. Board Membership: American Shoulder and Elbow Surgeons.

Any additional authors, their immediate families, and any research foundations with which they are affiliated have not received any financial payments or other benefits from any commercial entity related to the subject of this article.
