# Supplementary material for: Evaluating the safety of glucagon-like peptide-1 agonist use in non-diabetic overweight patients undergoing shoulder arthroplasty
Source: J Shoulder Elb Arthroplast. 2026 Sep 9;10(4):100088. doi: 10.1016/j.jsea.2026.100088 (PMC13675852; doi:10.1016/j.jsea.2026.100088)
Supplement: Supplementary file 1 [file mmc1.docx]

| **Appendix A** |  |
| --- | --- |
| **Procedure Type Codes** |  |
| TSA | CPT-23472, ICD-9-P-8180, ICD-10-P-0RRJ0JZ, ICD-10-P-0RRK0JZ |
| RTSA | ICD-9-P-8188, ICD-10-P-0RRJ00Z, ICD-10-P-0RRK00Z |
| GLP-1 Use | USC-39251, USC-18120 |
| **Exclusion Codes** |  |
| Infection | ICD-9-D-71101, ICD-9-D-71102, ICD-9-D-71111, ICD-9-D-71112, ICD-9-D-71121, ICD-9-D-71131, ICD-9-D-71122, ICD-9-D-71141, ICD-9-D-71142, ICD-9-D-71152, ICD-9-D-71151, ICD-9-D-71161, ICD-9-D-71162, ICD-9-D-71171, ICD-9-D-71172, ICD-9-D-71181, ICD-9-D-71182, ICD-9-D-71191, ICD-9-D-71192, ICD-9-D-73001, ICD-9-D-73002, ICD-9-D-73011, ICD-9-D-73012, ICD-9-D-73021, ICD-9-D-73022, ICD-10-D-M00019, ICD-10-D-M00219, ICD-10-D-M00819, ICD-10-D-M00029, ICD-10-D-M00129, ICD-10-D-M00229, ICD-10-D-M00829, ICD-10-D-M02319, ICD-10-D-M02329, ICD-10-D-M352, ICD-10-D-M352, ICD-10-D-M02119, ICD-10-D-M01X19, ICD-10-D-M01X29, ICD-10-D-M01X19, ICD-10-D-M01X29, ICD-10-D-M01X19, ICD-10-D-M01X29, ICD-10-D-M01X19, ICD-10-D-M01X29, ICD-10-D-M01X19, ICD-10-D-M01X29, ICD-10-D-M01X19, ICD-10-D-M01X29, ICD-10-D-M86119, ICD-10-D-M86219, ICD-10-D-M86129, ICD-10-D-M86229, ICD-10-D-M86619, ICD-10-D-M86629, ICD-10-D-M869, ICD-10-D-M869 |
| Pathologic | ICD-10-D-M84429A, ICD-10-D-M84421A, ICD-10-D-M84421D, ICD-10-D-M84421G, (ICD-10-D-M84421K - ICD-10-D-M84629S) , (ICD-10-D-S42201A - ICD-10-D-S42409S |
| Osteoporosis | ICD-9-D-73300:ICD-9-D-73303, ICD-9-D-73309, ICD-10-D-M810, ICD-10-D-M816, ICD-10-D-M818 |
| Inflammatory | ICD-9-D-7149, ICD-9-D-7140, ICD-9-D-6960, ICD-9-D-7169, ICD-9-D-71489, ICD-10-D-M05061, ICD-10-D-M05062, ICD-10-D-M05069, ICD-10-D-M05161, ICD-10-D-M05162, ICD-10-D-M05169, ICD-10-D-M05361, ICD-10-D-M05362, ICD-10-D-M05369, ICD-10-D-M05461, ICD-10-D-M05462, ICD-10-D-M05469, ICD-10-D-M05561, ICD-10-D-M05562, ICD-10-D-M05569, ICD-10-D-M05661, ICD-10-D-M05662, ICD-10-D-M05669, ICD-10-D-M05761, ICD-10-D-M05762, ICD-10-D-M05769, ICD-10-D-M05861, ICD-10-D-M05862, ICD-10-D-M05869, ICD-10-D-M059, ICD-10-D-M069, ICD-10-D-M06861, ICD-10-D-M06862, ICD-10-D-M06869, ICD-10-D-M06061, ICD-10-D-M06062, ICD-10-D-M06063, ICD-10-D-M10061, ICD-10-D-M10062, ICD-10-D-M10069, M11861, ICD-10-D-M11862, ICD-10-D-M11869 |
| Cancer | ICD-9-D-140, ICD-9-D-17299, ICD-9-D-174, ICD-9-D-195899, ICD-9-D-200, ICD-9-D-20899, ICD-9-D-2386, ICD-10-D-C00, ICD-10-D-C2699, ICD-10-D-C30, ICD-10-D-C3499, ICD-10-D-C37, ICD-10-D-C4199, ICD-10-D-C43, ICD-10-D-C4399, ICD-10-D-C45, ICD-10-D-C5899, ICD-10-D-C60, ICD-10-D-C7699, ICD-10-D-C81, ICD-10-D-C8599, ICD-10-D-C88, ICD-10-D-C8899, ICD-10-D-CC90, ICD-10-D-C9799, ICD-9-D-1960, ICD-9-D-1999, ICD-10-D-C770, ICD-10-D-C809 |
| Fracture | ICD-9-D-73311, ICD-9-D-81200, ICD-9-D-81201, ICD-9-D-81202, ICD-9-D-81203, ICD-9-D-81209, ICD-9-D-81210, ICD-9-D-81211, ICD-9-D-81212, ICD-9-D-81213, ICD-9-D-81219, ICD-9-D-81220, ICD-9-D-81221, ICD-9-D-81230, ICD-9-D-81231, ICD-9-D-81240, ICD-10-D-M84429A, ICD-10-D-M84421A, ICD-10-D-M84421D, ICD-10-D-M84421G, (ICD-10-D-M84421K - ICD-10-D-M84629S) , (ICD-10-D-S42201A - ICD-10-D-S42409S |
| Obesity | ICD-10-D-E6601, ICD-10-D-Z6840, ICD-10-D-Z6841, ICD-10-D-Z6842, ICD-10-D-Z6843, ICD-10-D-Z6844, ICD-10-D-Z6845, ICD-9-D-V8541, ICD-9-D-V8542, ICD-9-D-V8543, ICD-9-D-V8544, ICD-9-D-V8545, ICD-9-D-2780,1ICD-10-D-Z6835, ICD-10-D-Z6836, ICD-10-D-Z6837, ICD-10-D-Z6838, ICD-10-D-Z6839, ICD-9-D-V8535, ICD-9-D-V8536, ICD-9-D-V8537, ICD-9-D-V8538, ICD-9-D-V8539 |
|  |  |
| *2-Year Outcomes* |  |
| Fracture | ICD-9-D-73311, ICD-9-D-81200, ICD-9-D-81201, ICD-9-D-81202, ICD-9-D-81203, ICD-9-D-81209, ICD-9-D-81210, ICD-9-D-81211, ICD-9-D-81212, ICD-9-D-81213, ICD-9-D-81219, ICD-9-D-81220, ICD-9-D-81221, ICD-9-D-81230, ICD-9-D-81231, ICD-9-D-81240, ICD-10-D-M84429A, ICD-10-D-M84421A, ICD-10-D-M84421D, ICD-10-D-M84421G, ICD-10-D-M84421K, ICD-10-D-M84629S, ICD-10-D-S42201A, ICD-10-D-S42409S, ICD-9-D-81220, ICD-9-D-81200, ICD-9-D-81221, ICD-9-D-99644, ICD-10-D-M9731XA, ICD-10-D-M9732XA, ICD-10-D-S42209A, ICD-10-D-S42399A, ICD-9-D-81100, ICD-9-D-81101, ICD-9-D-81102, ICD-9-D-81103, ICD-9-D-81109, ICD-10-D-S42102A, ICD-10-D-S42101A, ICD-10-D-S42141A, ICD-10-D-S42142A, ICD-10-D-S42112A, ICD-10-D-S42109A, ICD-10-D-S42111A, ICD-10-D-S42192A, ICD-10-D-S42114A, ICD-10-D-S42115A, ICD-10-D-S42191A, ICD-10-D-S42144A, ICD-10-D-S42145A, ICD-10-D-S42143A, ICD-10-D-S42113A, ICD-10-D-S42116A, ICD-10-D-S42146A, ICD-10-D-S42151A, ICD-10-D-S42152A, ICD-10-D-S42153A, ICD-10-D-S42154A, ICD-10-D-S42155A, ICD-10-D-S42156A, ICD-10-D-S42199A, ICD-9-D-81101, ICD-10-S42121A, ICD-10-S42122A, ICD-10-S42123A, ICD-10-S42124A, ICD-10-S42125A, ICD-10-S42126A, ICD-9-D-81101, ICD-10-S42121A, ICD-10-S42122A, ICD-10-S42123A, ICD-10-S42124A, ICD-10-S42125A, ICD-10-S42126A |
| Dislocation | ICD-10-D-T84020A, ICD-10-D-T84020D, ICD-10-D-T84020S, ICD-10-D-T84021A, ICD-10-D-T84021D, ICD-10-D-T84021S, ICD-10-D-T84029A, ICD-10-D-T84029D, ICD-10-D-T84029S, ICD-9-D-99642, ICD-9-D-71831, ICD-9-D-83100, ICD-9-D-83101, ICD-9-D-99642, ICD-10-D-M24411, ICD-10-D-M24412, ICD-10-D-M24419, ICD-10-D-S43004A, ICD-10-D-S43005A, ICD-10-D-S43006A, ICD-10-D-S43084A, ICD-10-D-S43085A, ICD-10-D-S43086A, ICD-10-D-T84029A, ICD-10-D-S43016AICD-9-D-71831, ICD-9-D-83100, ICD-9-D-83101, ICD-9-D-99642, ICD-10-D-M24411, ICD-10-D-M24412, ICD-10-D-M24419, ICD-10-D-S43004A, ICD-10-D-S43005A, ICD-10-D-S43006A, ICD-10-D-S43084A, ICD-10-D-S43085A, ICD-10-D-S43086A, ICD-10-D-T84029A, ICD-10-D-S43016A |
| Stiffness | ICD-9-D-71851, ICD-9-D-71951, ICD-9-D-7260, ICD-10-D-M25611, ICD-10-D-M25612, ICD-10-D-M25619, ICD-10-D-M7500, ICD-10-D-M7501, ICD-10-D-M7502, ICD-10-D-M24611, ICD-10-D-M24612, ICD-10-D-M24619 |
| Loosening | ICD-9-D-73342, ICD-9-D-99641, ICD-9-D-99649, ICD-9-D-99643, ICD-10-D-T84038A, ICD-10-D-T84039A, ICD-10-D-T84019A, ICD-9-D-99647, ICD-9-D-99649 |
| PJI | ICD-9-D-71101, ICD-9-D-71102, ICD-9-D-71111, ICD-9-D-71112, ICD-9-D-71121, ICD-9-D-71131, ICD-9-D-71122, ICD-9-D-71141, ICD-9-D-71142, ICD-9-D-71152, ICD-9-D-71151, ICD-9-D-71161, ICD-9-D-71162, ICD-9-D-71171, ICD-9-D-71172, ICD-9-D-71181, ICD-9-D-71182, ICD-9-D-71191, ICD-9-D-71192, ICD-9-D-73001, ICD-9-D-73002, ICD-9-D-73011, ICD-9-D-73012, ICD-9-D-73021, ICD-9-D-73022, ICD-10-D-M00019, ICD-10-D-M00219, ICD-10-D-M00819, ICD-10-D-M00029, ICD-10-D-M00129, ICD-10-D-M00229, ICD-10-D-M00829, ICD-10-D-M02319, ICD-10-D-M02329, ICD-10-D-M352, ICD-10-D-M352, ICD-10-D-M02119, ICD-10-D-M01X19, ICD-10-D-M01X29, ICD-10-D-M01X19, ICD-10-D-M01X29, ICD-10-D-M01X19, ICD-10-D-M01X29, ICD-10-D-M01X19, ICD-10-D-M01X29, ICD-10-D-M01X19, ICD-10-D-M01X29, ICD-10-D-M01X19, ICD-10-D-M01X29, ICD-10-D-M86119, ICD-10-D-M86219, ICD-10-D-M86129, ICD-10-D-M86229, ICD-10-D-M86619, ICD-10-D-M86629, ICD-10-D-M869, ICD-10-D-M869 |
| Revision | CPT-23473, CPT-23474, ICD-9-P-8197, ICD-10-P-0RWJ0JZ, ICD-10-P-0RWJ4JZ, ICD-10-P-0RWK0JZ |
| Osteolysis | ICD-10-D-T84090A, ICD-10-D-T84090D, ICD-10-D-T84090S, ICD-10-D-T84091A, ICD-10-D-T84091D, ICD-10-D-T84091S, ICD-10-D-T84099A, ICD-10-D-T84099D, ICD-10-D-T84099S |
| *90-Day Outcomes* |  |
| DVT | ICD-10-D-I8240, ICD-10-D-I82401, ICD-10-D-I82402, ICD-10-D-I82403, ICD-10-D-I82409, ICD-10-D-I82410, ICD-10-D-I82411, ICD-10-D-I82412, ICD-10-D-I82413, ICD-10-D-I82419, ICD-10-D-I82420, ICD-10-D-I82421, ICD-10-D-I82422, ICD-10-D-I82423, ICD-10-D-I82429, ICD-10-D-I824Y1, ICD-10-D-I824Y2, ICD-10-D-I824Y3, ICD-10-D-I824Y9, ICD-10-D-I824Z1, ICD-10-D-I824Z2, ICD-10-D-I824Z3, ICD-10-D-I824Z9, ICD-10-D-I82431, ICD-10-D-I82432, ICD-10-D-I82433, ICD-10-D-I82439, ICD-10-D-I82441, ICD-10-D-I82442, ICD-10-D-I82443, ICD-10-D-I82449, ICD-10-D-I82451, ICD-10-D-I82452, ICD-10-D-I82453, ICD-10-D-I82459, ICD-10-D-I82461, ICD-10-D-I82462, ICD-10-D-I82463, ICD-10-D-I82469, ICD-10-D-I82491, ICD-10-D-I82492, ICD-10-D-I82493, ICD-10-D-I82499, ICD-10-D-I82621, ICD-10-D-I82622, ICD-10-D-I82623, ICD-10-D-I82629 |
| PE | ICD-10-D-I2600, ICD-10-D-I2602, ICD-10-D-I2609, ICD-10-D-I2699 |
| Death | ICD-9-D-7980, ICD-9-D-7981, ICD-9-D-7982, ICD-9-D-7989, ICD-10-D-R99 |
| SSI | ICD-10-D-L0889, ICD-10-D-L089, ICD-10-D-T814XXA, ICD-10-D-L03119, ICD-10-D-L03129, ICD-10-D-L03319, ICD-10-D-L03329 |
| ED Visits | CPT-99281:CPT-99285 |
| *Wound disruption, acute kidney injury, hematoma, nerve Injury, pneumonia, transfusion, urinary tract infection, readmission were all encoded by the database | |
